# A Two-Stage Model for Lipid Modulation of the Activity of Integral Membrane Proteins

**DOI:** 10.1371/journal.pone.0039255

**Published:** 2012-06-19

**Authors:** Martín M. Dodes Traian, Diego I. Cattoni, Valeria Levi, F. Luis González Flecha

**Affiliations:** 1 Laboratorio de Biofísica Molecular – Instituto de Química y Fisicoquímica Biológicas, Universidad de Buenos Aires - CONICET, Buenos Aires, Argentina; 2 Laboratorio de Dinámica Intracelular– Departamento de Química Biológica, Facultad de Ciencias Exactas y Naturales, Universidad de Buenos Aires, Buenos Aires, Argentina; 3 Centre de Biochimie Structurale, INSERM U554, CNRS UMR 5048, Université de Montpellier 1 and 2, Montpellier, France; University of Cambridge, United Kingdom

## Abstract

Lipid-protein interactions play an essential role in the regulation of biological function of integral membrane proteins; however, the underlying molecular mechanisms are not fully understood. Here we explore the modulation by phospholipids of the enzymatic activity of the plasma membrane calcium pump reconstituted in detergent-phospholipid mixed micelles of variable composition. The presence of increasing quantities of phospholipids in the micelles produced a cooperative increase in the ATPase activity of the enzyme. This activation effect was reversible and depended on the phospholipid/detergent ratio and not on the total lipid concentration. Enzyme activation was accompanied by a small structural change at the transmembrane domain reported by 1-aniline-8-naphtalenesulfonate fluorescence. In addition, the composition of the amphipilic environment sensed by the protein was evaluated by measuring the relative affinity of the assayed phospholipid for the transmembrane surface of the protein. The obtained results allow us to postulate a two-stage mechanistic model explaining the modulation of protein activity based on the exchange among non-structural amphiphiles at the hydrophobic transmembrane surface, and a lipid-induced conformational change. The model allowed to obtain a cooperativity coefficient reporting on the efficiency of the transduction step between lipid adsorption and catalytic site activation. This model can be easily applied to other phospholipid/detergent mixtures as well to other membrane proteins. The systematic quantitative evaluation of these systems could contribute to gain insight into the structure-activity relationships between proteins and lipids in biological membranes.

## Introduction

Initially thought as a structural anchor for proteins and a barrier to separate the cell from its environment, our view of the cell membrane has largely evolved since the original description of the fluid mosaic model [1]. Nowadays, we know that membranes are not an inert support for membrane proteins but have an essential role in determining and regulating their function [2]. Interactions between membrane proteins and membrane lipids can be grouped into two main categories. The first one includes lipid molecules that appear resolved in high resolution structures of membrane proteins interacting at specific sites [3], [4]. These structural lipids are not removed during membrane solubilization and the extensive detergent washing performed during membrane protein purification [5]. The second category includes phospholipids that can interact with the transmembrane surface of membrane proteins at non-specific sites. This generates a monolayer of phospholipids with a restricted mobility compared to that of bulk lipids [6] but that can rapidly exchange with characteristic times in the range of 10^−8^–10^−4^ s [7], [8]. Both types of interactions are critical for the structure and function of membrane proteins [9]. However, the molecular mechanisms underlying this regulation remain unclear.

The erythrocyte plasma membrane Ca^2+^ pump (PMCA) is a very good model to study the effects of phospholipids on membrane protein function. PMCA is an integral helical membrane protein consisting of a single polypeptide chain with a molecular mass of 134 kDa [10]. A large intracellular domain embraces the catalytic and regulatory sites [11] while short external loops connect 10 transmembrane segments organized in 3 hydrophobic clusters [12], [13]. Its enzymatic activity has been thoroughly characterized in its native environment [11]. When the protein is purified from detergent-solubilized erythrocyte plasma membranes and reconstituted in phospholipid-detergent mixed micelles, it preserves the biochemical properties of the enzyme in the erythrocyte membrane [14] which has validated the use of these micellar preparations for structural and functional studies [15]. It has been shown that PMCA activity and stability are influenced by phospholipids [16], [17]. Particularly, neutral phospholipids like phosphatidylcholine and phosphatidylethanolamine produce what is known as the basal activity of the enzyme, whereas acidic phospholipids like phosphatidylserine and phosphatidylinositol act as specific activators [18], [19], [20].

In this work we explore the regulation of basal PMCA activity in mixed micelles of 1,2-dipalmitoyl-sn-glycero-3-phosphocholine (DPPC) and the detergent poly(oxyethylene)10-lauryl ether (C_12_E_10_). The obtained results allow us to postulate a two-stage mechanistic model that explains this modulation based on the exchange among non-structural amphiphiles at the transmembrane surface of the protein, and a small lipid-induced conformational change.

## Results and Discussion

### Micelle Composition Modulates the ATPase Activity of PMCA

**Figure 1:**
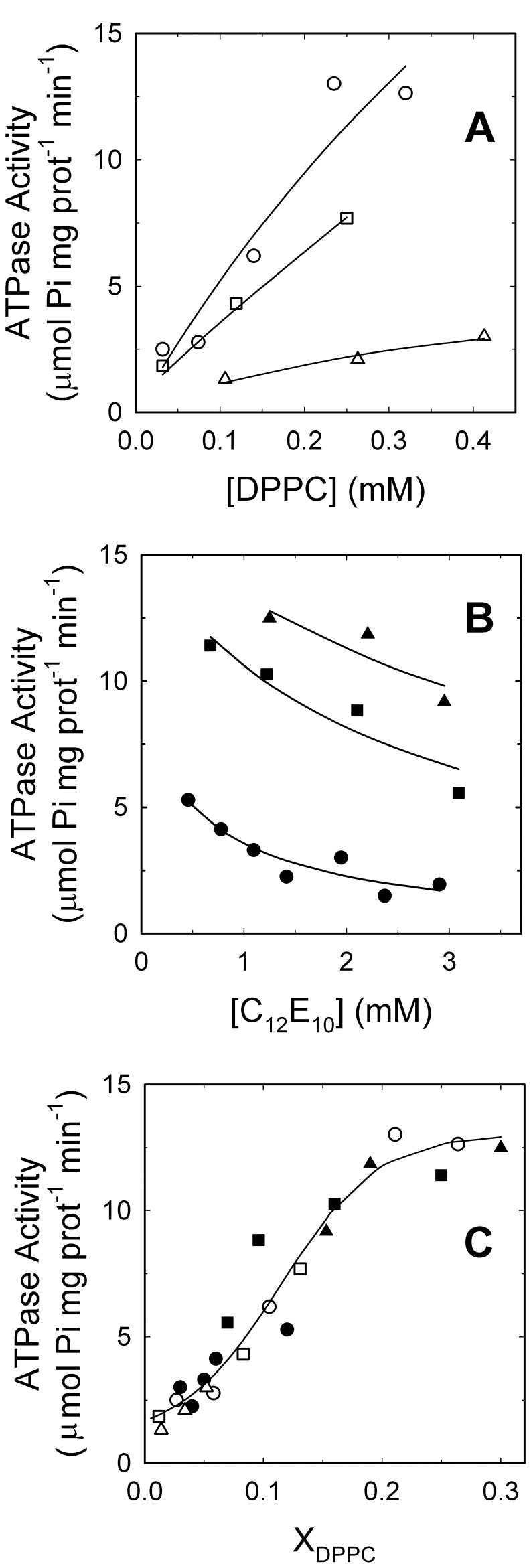
Effects of phospholipids and detergent on the ATPase activity of PMCA. (A) Purified PMCA, reconstituted in (○) 1.20, (□) 2.75 and (▵) 7.50 mM C_12_E_10_ micelles was supplemented with increasing concentrations of DPPC before measuring the ATPase activity. (B) Alternatively, C_12_E_10_ was added to purified PMCA already supplemented with (•) 0.06, (▪) 0.22 and (▴) 0.53 mM DPPC before measuring the ATPase activity. Solid lines in A and B are a guide to the eye. (C) Data from panels A and B were plotted as a function of the phospholipid mole fraction in the micellar phase. The continuous line in C corresponds to the graphical representation of equation 8 with the best fitting parameter values shown in Table 1.

To evaluate the regulation of basal PMCA activity by phospholipids and detergents, the ATPase activity of the purified protein reconstituted in detergent micelles was assessed by following two different protocols. First by assaying the effect of increasing DPPC concentration while keeping constant the concentration of C_12_E_10_ (Fig. 1A), and second by evaluating the effects of increasing C_12_E_10_ concentration leaving DPPC concentration at fixed values (Fig. 1B).

**Table 1:** Model parameters estimated by global fitting.

Parameter	Value
*A*_0_	1.1±0.9 µmol Pi • mg prot^−1^ • min^−1^
*A*_1_	12.8±0.8 µmol Pi • mg prot^−1^ • min^−1^
θ_0.5_	0.16±0.02
*c*_θ_	15±4
*K*_ex,DPPC_	1.4±0.5
*ξ*	51±10

When lipid-depleted PMCA was incubated in the activity medium without adding DPPC, the ATPase activity was 2.3±0.2 µmol Pi mg PMCA^−1^ min^−1^. In this condition only structural lipids might be present and could give account of this low activity. Figure 1A shows that addition of DPPC greatly increased the ATPase activity following different activation isotherms depending on the total C_12_E_10_ concentration. On the contrary, Figure 1B shows that at fixed DPPC concentrations PMCA activity decreases when increasing C_12_E_10_ concentration; being this inactivation effect more pronounced for the lower phospholipid concentrations. It is important to mention that the enzyme purified with C_12_E_10_ fully recovers its ATPase activity by addition of phospholipids, reaching values comparable to those of the enzyme purified in the presence of mixed phospholipid/detergent micelles [15], [21], [22]. These results indicate that the modulation of the enzyme activity by phospholipids and non-denaturing detergents is completely reversible.

The data presented in Figures 1A and 1B were re-plotted as a function of phospholipid mole fraction in the micellar phase (Fig. 1C). Given their low critical micellar concentration (∼ 0.5 nM for DPPC [23] and ∼ 5 µM for C_12_E_10_ [16]), we assumed that both detergent and phospholipid molecules are exclusively present in the micelles. Figure 1C shows that a single trend line was now able to describe the full set of experiments indicating that the observed behaviour is directly related to the composition of the micellar phase and not to the total concentration of the individual amphiphiles.

### Phospholipid Activation of PMCA is Accompanied by Small Structural Changes

To monitor possible conformational transitions of PMCA associated to the phospholipid-induced activation described in the previous section, its overall structure was explored by using different spectroscopic techniques.

**Figure 2:**
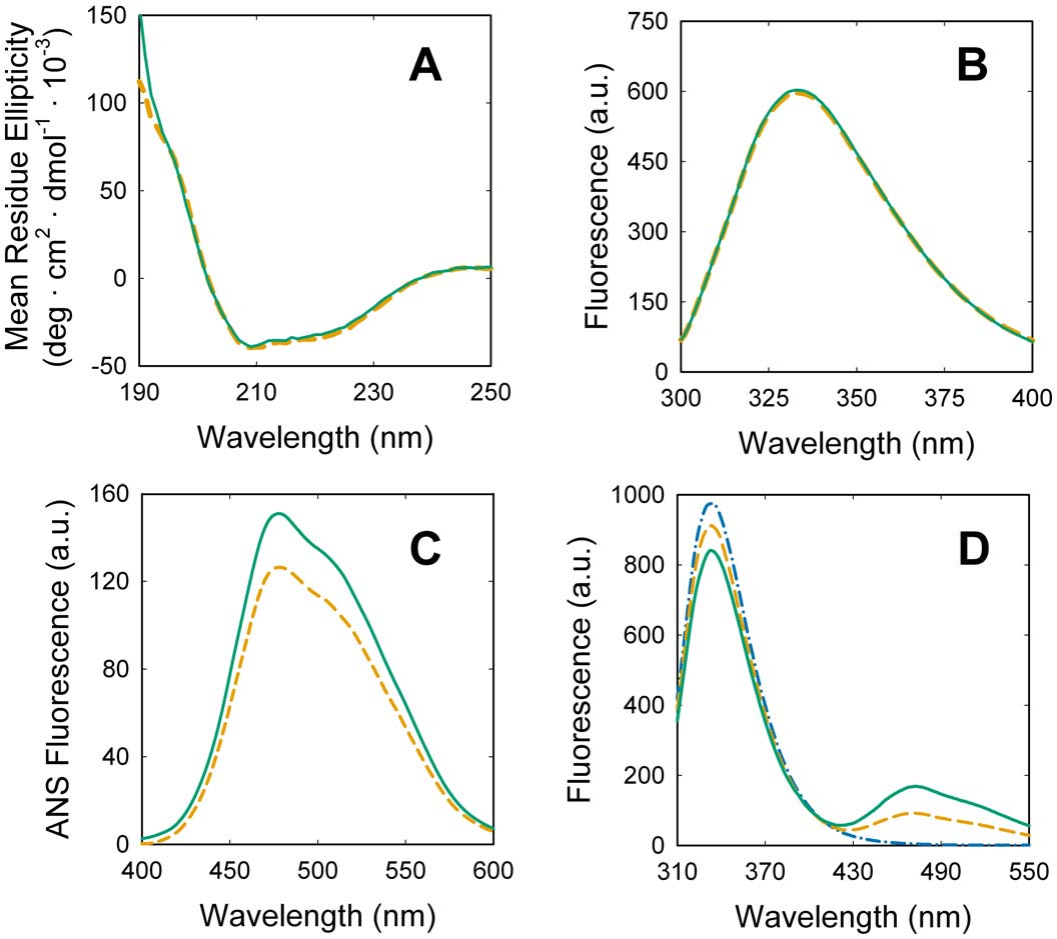
PMCA structural changes upon activation by DPPC. Purified PMCA was supplemented with C_12_E_10_ up to 1.2 mM (orange dashed lines) or up to 1.7 mM DPPC/C_12_E_10_ and a final DPPC mole fraction of 0.3 ([C_12_E_10_] = 1.2 mM) (continuous green lines). After 10 minutes of incubation at 25°C, (A) far UV circular dichroism, and (B) Trp fluorescence were registered. ANS and Trp fluorescence were registered after adding 3 µM ANS to both samples and exciting at 380 nm (ANS, C) and at 295 nm (PMCA-ANS FRET, D). The emission spectrum of PMCA in the absence of ANS is shown as blue dash dotted line. Apparent energy transfer efficiencies were 0.14 in the presence of lipids and 0.07 in the absence of lipids. The final volume and protein concentration in all the samples was identical in order to avoid dilution corrections.

Far UV circular dichroism (CD) allows exploring secondary structure of proteins. The far UV CD spectrum of lipid-free PMCA (Fig. 2A) shows the main spectral characteristic expected for a helical membrane protein [24], [25], [26]. The addition of DPPC did not produce significant changes in the spectrum of PMCA, suggesting that phospholipid activation does not involve major changes in the overall secondary structure of the protein.

To further explore the structural perturbations responsible for phospholipid activation of PMCA, two fluorescent probes reporting on the status of hydrophobic regions were used: intrinsic Trp residues and the extrinsic probe 1-aniline-8-naphtalenesulfonate (ANS). Trp residues in membrane proteins are preferentially located at the water/membrane interface [27], allowing to monitor changes in the protein tertiary structure involving this region. The fluorescence spectrum of lipid-free PMCA is centered at 335 nm as expected for folded membrane proteins [25], [28], [29] and did not present significant changes after the addition of DPPC (Fig. 2B). This result indicates that no major changes are revealed by Trp at the transmembrane domain of PMCA after addition of phospholipids. The fluorescent probe ANS was widely used for the study of conformational transitions involving hydrophobic cavities in proteins [30], [31]. Only few native proteins bind ANS in hydrophobic pockets, including membrane proteins [21], [25], [32] for which ANS represents a useful reporter of the transmembrane domain. It can be observed that ANS fluorescence increases after adding DPPC to lipid-free PMCA (Fig 2C), suggesting a change in the environment from which ANS is emitting. The relationship between the increase in ANS fluorescence and changes in protein conformation is well known for water-soluble proteins; however the significance of similar changes in membrane proteins is less clear. Being ANS a hydrophobic probe, it also exhibits fluorescence when located in the hydrophobic core of micelles, therefore control experiments are necessary to assure that signal changes come from the protein**.** Fluorescence of ANS was registered in protein-free detergent and lipid-detergent micelles, and it was equal in both systems independently of the phospholipid mole fraction. Thus, the fluorescence increase observed in Figure 2C can be assigned to structural changes in the protein after lipid addition. On the other hand, Förster resonance energy transfer (FRET) between Trp and ANS has been previously used to monitor several protein processes [31]. This approach is particularly useful for membrane proteins because free micelles loaded with ANS do not display fluorescence in the spectral region corresponding to Trp emission. Figure 2D shows the fluorescence emission spectra obtained for PMCA solubilized in detergent micelles in the presence of ANS. The addition of DPPC up to a mole fraction that produced maximal PMCA activation, induced an increase in the maximal fluorescence of ANS (peak at ∼475 nm) concomitantly with a decrease in Trp fluorescence (peak at ∼335 nm) indicating a higher FRET efficiency. This result indicates that the average distance between the hydrophobic pockets reported by ANS and the Trp residues located at the water/lipid interface decreased after adding phospholipids, suggesting a closer packing of the transmembrane domain of PMCA upon activation.

### A Two-stage Model for Lipid Modulation of PMCA Activity

**Figure 3:**
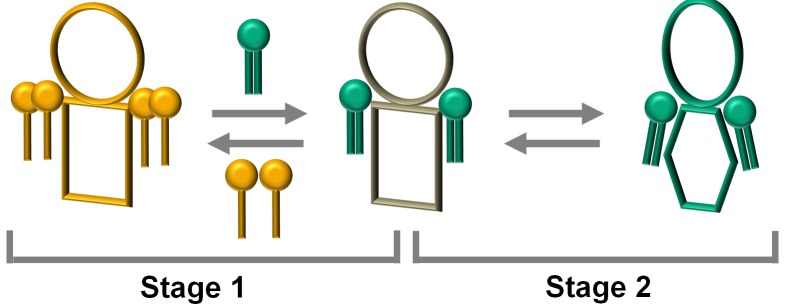
The two stage model for lipid modulation of the enzyme activity. The scheme shows the transition between low and high activity states of PMCA. In the first stage the enzyme selects a particular lipidic microenvironment among the available amphiphiles according to their relative affinities. The interaction of the protein with specific phospholipids induces, in a second stage, a conformational change at the transmembrane region which is further propagated towards the catalytic domain.

In the previous sections we showed that addition of small amounts of phospholipids to detergent solubilized PMCA produced a significant increase in its ATPase activity accompanied by a small conformational change. In this context, we propose a two-stage model accounting for PMCA activity regulation (Fig. 3). Initially, given the different relative affinities of the amphiphiles for the transmembrane region of the protein, a specific lipidic microenvironment with a different composition from the bulk micelar phase is generated (Fig. S1). The second stage is characterized by a subtle conformational change occurring at the transmembrane region and propagated towards the catalytic domain.

The first stage is supported by extensive evidence on the existence of a population of motionally-restricted amphiphiles surrounding the transmembrane region of membrane proteins ([33] and references therein). For this analysis we first define θ_DPPC_ as the fraction of the protein transmembrane surface covered by DPPC:
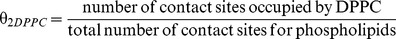
(1)Assuming a simple Langmuir-type adsorption model [34] it can be demonstrated that θ_DPPC_ is related to the DPPC mole fraction (*X*_DPPC,mic_) as [35]:
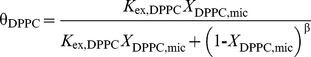
(2)where β is a stoichiometric coefficient for the phospholipid/detergent exchange that is equal to 2 for the amphiphiles employed in this study [35], and the exchange constant *K_ex,DPPC_*, results from the combination of the adsorption and desorption rate coefficients of the amphiphiles (*k_ad_* and *k_d_*, respectively):
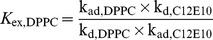
(3)Therefore, *K_ex,DPPC_* gives a measure of the affinity of DPPC, respect to a reference amphiphile (C_12_E_10_ in this study), for the hydrophobic transmembrane surface of the protein.

**Figure 4:**
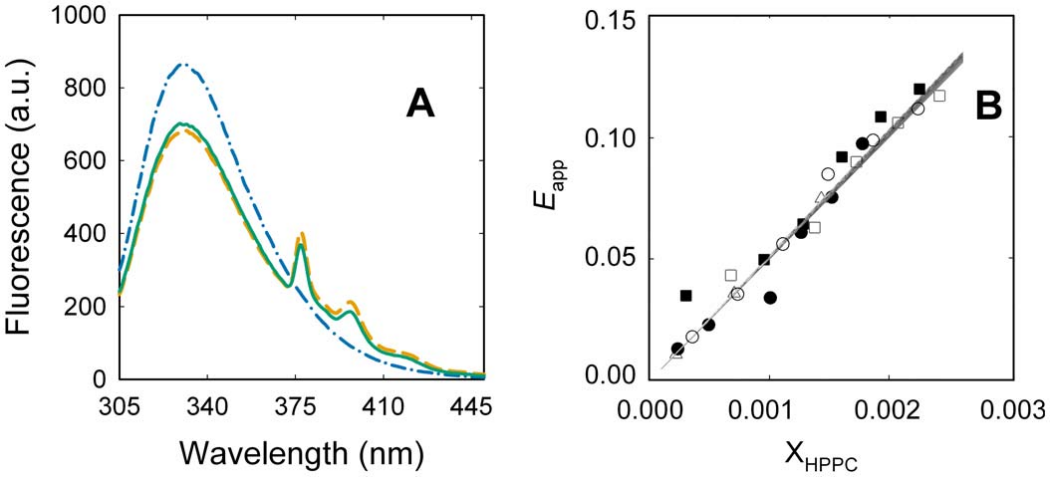
Determination of the exchange constant between DPPC and C_12_E_10_. (A) Lipid free purified PMCA (blue dash dotted line), PMCA with HPPC (orange dashed line) and PMCA-HPPC with DPPC/C_12_E_10_ up to a final DPPC mole fraction of 0.3 (continuous green line) were excited at 295 nm and fluorescence emission spectra were registered. (B) PMCA samples were supplemented with DPPC up to mole fractions of: 0.34 (▵), 0.36 (•), 0.41(▪), 0.44 (□), 0.48 (○). PMCA emission intensity was measured after adding increasing quantities of HPPC and mixing for 1 min. Total intensity values were corrected for the dilution (<7%) caused by the addition of the probe. The 2D projection surface is the graphical representation of equation 4 with the best fitting parameter values shown in Table 1.

The exchange constant can be accurately measured using a FRET-based assay with a pyrene labeled phosphatidylcholine probe (1-hexadecanoyl-2-(1-pyrenedecanoyl)-sn-glycero-3-phosphocholine, HPPC) monitoring the exchange among the unlabeled amphiphiles [35], [36]. Figure 4A shows the fluorescence spectra of PMCA obtained in the absence and presence of HPPC. It can be observed that the addition of HPPC produces a significant decrease in Trp fluorescence, indicating the presence of HPPC molecules within the Förster distance from Trp residues. The subsequent addition of DPPC up to a mole fraction that produced maximal PMCA activation induced a small increase in PMCA fluorescence indicating a lower FRET efficiency in this latest condition (Fig. 4A, green line). This result contrast with that reported by ANS-Trp FRET experiments (Fig. 2D), and can be explained by the displacement of the fluorescent lipid by DPPC on the transmembrane surface of PMCA [35], [36].

The apparent energy transfer efficiency (*E*_app_) measured in each condition (Fig 4B) is related to the micellar mole fraction of HPPC (*X*_HPPC,mic_) and that of unlabeled phospholipids (*X*_DPPC,mic_) as [35]:
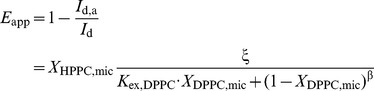
(4)where *I*_d_ and *I*_d,a_ represent the intensity of Trp fluorescence in the absence or presence of the probe respectively, and ξ is a parameter defined by the protein/probe pair. The exchange constant between DPPC and C_12_E_10_ can be determined by fitting equation 4 to the experimental data on Figure 4B. The obtained value (1.4±0.4) indicates that the affinity of DPPC for the transmembrane surface of PMCA is around 50% higher than that of the detergent.

The composition of the boundary monolayer of motionally-restricted amphiphiles surrounding the transmembrane domain of the protein, i.e. the effective microenvironment sensed by the protein, can be determined for a given *K*_ex_ value using equation 2. Figure S1 shows a sigmoidal relationship between the boundary monolayer composition and the micelle composition. This could explain the cooperative effect observed in Figure 1C. However**,** it can be noted that the sigmoidal character of phospholipid activation is still present when the activity is plotted as a function of the fractional coverage of the hydrophobic transmembrane surface of PMCA by phospholipids (Fig S2), indicating that this is an intrinsic property of the phospholipid activation effect. This cooperative-like behaviour can be empirically described by a differential logistic function [37],
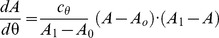
(5)


In this equation *A* represents the measured catalytic activity of PMCA while *A*_o_ and *A*_1_ are the minimal and maximal asymptotic activities. The parameter *c*_θ_ represents an empirical cooperativity coefficient related to the steepest relative change in the enzyme activity. This maximal slope is reached when *A* = (*A*_1_–*A*_o_)/2, i.e. at half-maximal activation (Fig. S3A). The composition of the boundary monolayer at this point is denoted as θ_0.5_ (Fig. S3B).

It can be seen from equation 5 that:
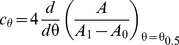
(6)


In terms of our model, this parameter gives a measure of the efficiency of the transduction step mediating between the sensing of the phospholipidic environment and the enzymatic catalysis.

Integrating equation 5, gives:
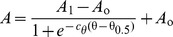
(7)and replacing equation 2 in 7 we obtain:



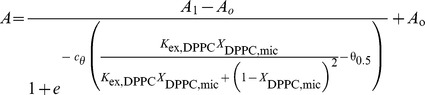
(8)Equation 8 describes the dependence of PMCA catalytic activity on the composition of the micellar phase where the enzyme is reconstituted.

The parameter *K*_ex_,_DPPC_ plays an important role in this model since it allows to link the experiments shown in Figures 1C and 4B. Thus, the best estimation of the parameters defining the proposed model was obtained by global fitting of equations 8 and 4 to the complete set of experimental data presented in this study (Table 1). The continuous line in Figure 1C, and the two-dimensional projection plot in Figure 4B show that the model accurately describes the whole set of independent experiments. Moreover, the small and randomly distributed residuals confirmed the quality of the fit (Fig. S4). The obtained results show that DPPC activation of PMCA is characterized by an 11-fold increase in the minimal asymptotic ATPase activity of PMCA, a half-maximal activation is observed when 16% of the transmembrane surface of PMCA is covered by DPPC, and starting from this last condition, the empirical cooperativity coefficient indicates that a 4% increase in the DPPC coverage is transduced in a 15% increase in the ATPase activity.

### A Minimal Model for Describing the Phospholipid Effects on the Enzymatic Activity of Integral Membrane Proteins

The two-stage model presented in the previous section accurately describes the PMCA activation by DPPC and the change in the apparent energy transfer efficiency between PMCA and a fluorescent lipid by the addition of DPPC. Despite experimentally assessing lipid-protein interactions is possible by using the FRET approach described in this work, or other spectroscopic methodologies (e.g. EPR [7], fluorescence quenching [38]), for some membrane proteins these measurements can represent a significant challenge. In those cases it could be rather useful to have an alternative and simplified model to study only the activation effect by phospholipids. This model shall include the same information than the two-stage model, but in one minimal set of new parameters. Again, a logistic function (Eq. 9), now being a function of *X*_DPPC_, was the best suited to give account of the observed activation effect.
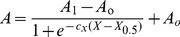
(9)where *X*_0.5_ represents the phospholipid mole fraction that gives half activation effect, and *c*_x_ the empirical cooperativity coefficient related to the maximal slope in the plot on Fig 1C. Fitting equation 9 to the experimental data allowed obtaining the best fitting values of these parameters (Table 2).

**Table 2:** Minimal model best fit parameter values.

Parameter	Value
*A*_0_	1.6±0.6 µmol Pi • mg prot^−1^ • min^−1^
*A*_1_	13±1 µmol Pi • mg prot^−1^ • min^−1^
*X*_0.5_	0.12±0.01
*c*_x_	26±6

To further explore the relationship among the parameters of the minimal model and those of the two-stage model we performed a series of simulations of activity data by using equation 8 while varying the value of one of the parameters at a time. Equation 9 was then fitted to each series of simulated data and the best fit values of the parameters of the minimal model were plotted as a function of the two-stage model parameter being evaluated (Fig. S5).

It can be observed that when keeping constant the parameters corresponding to the second stage (*c*_θ_ and θ_0.5_) both *c*_x_ and *X*_50_ are dependent on the value of *K*_ex,DPPC_ (Fig. S5. A and B). Interestingly, *c*_x_ displays a linear dependency on *K*_ex,DPPC_ indicating that in this situation the maximal slope of the activation curve gives a measure of the affinity of phospholipids for the transmembrane surface of the protein. On the other hand, when the parameter characterizing the first stage (*K*_ex,DPPC_) is kept constant *c*_x_ displays a cuasi-linear dependence on *c*_θ_ (Fig. S5 F) and *X*_0.5_ on θ_0.5_ (Fig. S5 C). The cross-dependencies (Fig. S5 D and E) were small in magnitude.

### Concluding Remarks

Micelles have been widely used as membrane-mimicking systems allowing to reconstitute membrane proteins in vitro while retaining its function and structure. Furthermore, recent studies reviewing the tolerance of membrane proteins to membrane lipid composition reinforce the validity of such model membranes and its relevance as source of valuable information [39]. In this work we have explored the regulation of the basal activity of the plasma membrane calcium pump reconstituted in phospholipid/detergent mixed micelles, demonstrating that it requires a minimum amount of lipids surrounding its transmembrane region to be fully active. The lipid-induced activation of PMCA is a reversible process that includes the exchange among non-structural lipids and minor conformational changes in the protein. This amphiphile-induced structural rearrangement should be initially restricted to the transmembrane region of the protein, but it has to be further propagated towards the catalytic domain to produce either activation or inhibition of the enzyme.

Hitherto, there is a lack of quantitative information about the effects of lipids on the biological activity of integral membrane proteins. This is a main limitation to understanding the molecular mechanisms by which lipid-protein interactions are determinant of the biological function of these proteins. Here we propose a simple two-stage model that can accurately describe the effects of DPPC on the enzymatic activity of PMCA, and can be easily applied to other lipids and other membrane proteins where cooperative-like lipid activation is observed (e.g. [40]). The model allowed us to obtain values of some key parameters with a clear physical meaning: The asymptotic minimal and maximal activities of the studied membrane protein, the degree of lipid coverage of the transmembrane surface producing half maximal activation, the relative affinity of a lipid for the hydrophobic transmembrane surface, and the efficiency of the transduction step relating lipid coverage and catalytic site activation. The systematic evaluation of these parameters for membrane proteins and lipids could contribute to shed light on the mechanism underlying the activation process and to gain insight into the structure-activity relationships between proteins and lipids in biological membranes.

## Materials and Methods

### Reagents

Poly(oxyethylene)10-lauryl ether (C_12_E_10_) and 1,2-dipalmitoyl-*sn*-glycero-3-phosphocholine (DPPC), were purchased from Sigma Chemical (St. Louis, MO) and Avanti Lipids (Alabaster, AL). The fluorescent probes 1-anilinonaphthalene-8-sulfonic acid (ANS) and 1-hexadecanoyl-2-(1-pyrenedecanoyl)-sn-glycero-3-phosphocholine (HPPC) were purchased from Molecular Probes (Eugene, OR). All other chemicals used in this work were of analytical grade.

### Purification of Plasma Membrane Ca^2+^ Pump from Human Erythrocytes

Human calmodulin-depleted erythrocyte membranes were prepared as described previously [41], [42]. Membranes (6–8 mg total protein/ml) were incubated 10 min at 4°C in extraction buffer with 7.8 mM C_12_E_10_ and 20% v/v glycerol, and then centrifuged at 20000×g for 30 min. PMCA was purified by affinity chromatography in a calmodulin-agarose column as described previously [22] but phospholipids were omitted in all the purification steps and replaced by 20% v/v glycerol. This method ensures almost complete delipidation of PMCA. Fractions exhibiting the highest Trp fluorescence and specific Ca^2+^-ATPase activity were pooled. Protein purity, integrity and concentration were evaluated by SDS-PAGE [43] using bovine serum albumin as standard. The final preparation, 500 nM PMCA, 80 µM C_12_E_10_ 130 mM KCl, 1 mM MgCl_2_, 2 mM EDTA, 2 mM CaCl_2_, 2 mM DTT, 20 mM MOPS (pH 7.4 at 4°C) and 20% v/v glycerol, was stored under liquid nitrogen until use.

### Preparation of Phospholipid-detergent Mixed Micelles

Phospholipids were solubilized in 1% C_12_E_10_ by vortexing the mixture above the transition temperature of DPPC (41°C), followed by sonication and sedimentation of non-solubilized material for 10 min at 12000×g. Phospholipid concentration was measured after mineralization of dried samples suspended in 50 µl of 72% perchloric acid for 40 min at 150–190°C. Mineralized samples were supplemented with 250 µl of 0.5 M NaOH and 800 µl H_2_O, and the released Pi was determined by the Malachite Green procedure [39], [40]. Solutions with different DPPC/C_12_E_10_ mole ratios were prepared by mixing adequate amounts of C_12_E_10_ stock solution and DPPC stock solution. Detergent concentration of the stock solution was determined by measuring the refractive index of the solution considering dn/dc = 0.11 ml/g for C_12_E_10_ [44].

### Measurement of Ca^2+^-ATPase Activity

ATPase activity was measured at 37°C as the initial rate of Pi release from ATP hydrolysis as described previously [21]. The activity medium was: 7 nM PMCA, 120 mM KCl; 30 mM MOPS-K (pH 7.4); 4 mM MgCl_2_; 1 mM EGTA; 1.1 mM CaCl_2_; 2 mM ATP and the phospholipid and C_12_E_10_ concentration are indicated for each experiment. The rate of ATP hydrolysis in the absence of Ca^2+^ was identical to that obtained in the same medium without the enzyme. The concentration of free Ca^2+^ was 140 µM and it was determined using an Orion 9320 ion-selective Ca^2+^ electrode. Pi release was estimated by Malachite Green Assay [45].

### Fluorescence Spectroscopy

Steady state fluorescence measurements were performed at 25°C in a 3×3 mm quartz cuvette using a Jasco FP-6500 spectrofluorimeter equipped with a Jasco ETC-237T peltier temperature controller. Both excitation and emission bandwidths were set at 3 nm. PMCA emission spectrum was registered between 305 and 400 nm after excitation at 295 nm. ANS fluorescence was registered between 400 and 600 nm following excitation at 380 nm and between 310 and 550 nm following excitation at 295 nm. Micellar fluorescence control was performed by registering the emission from the purification buffer with 1200 µM C_12_E_10_ and from the same buffer with 1700 µM DPPC/C_12_E_10_ (DPPC mole fraction = 0.3, [C_12_E_10_] = 1200 µM]. The concentration of micelles was adjusted to ensure full partitioning of ANS in the micelles measuring the partition constant of ANS into micelles (1490±40) as described [46]. The background emission of buffers (less than 5% of total fluorescence) was subtracted from all the spectra.

### Determination of the Exchange Constant between Phospholipids and Detergent

The relative affinity DPPC/C_12_E_10_ for the hydrophobic transmembrane surface of PMCA was determined according to Levi et al [35].

### Circular Dichroism

Circular dichroism spectra of PMCA were registered at 25°C in the wavelength region of 190–250 nm as was described [26] using a Jasco J-810 spectropolarimeter. Data were collected in a 1 mm path length cuvette using a scan speed of 20 nm/min with a time constant of 1 s. An average of three independent measurements was used to calculate the mean residue ellipticity as described [21].

### Data Analysis

Data presented in this work are representative of at least two independent experiments. Activity measurements were performed in duplicate or triplicate. Equations were fitted to the experimental data using a non-linear regression procedure based on the Gauss-Newton algorithm. The dependent variable was assumed to be homoscedastic (constant variance), and the independent variable was considered to have negligible error [47]. A weighted least squares procedure was used for global fitting.

## Supporting Information

Figure S1 **Dependence of the fractional coverage of the hydrophobic transmembrane surface of PMCA on the phospholipid mole fraction.** Simulated values of the fractional coverage of the transmembrane surface by phospholipids (θ_PL_) were obtained for the full range of micelle compositions using equation 2 and the *K*_ex_ values indicated in the figure. The stoichiometric coefficient was taken equal to 2. The orange line corresponds to the *K*_ex_ value determined in this work for the exchange DPPC/C_12_E_10_.
(TIF)

Figure S2 **ATPase activity dependence on the phospholipid fractional coverage of the hydrophobic transmembrane surface of PMCA.** Data shown in Figure 1C was represented as a function of the composition of the lipid boundary monolayer taken from Figure S1. The continuous line is the graphical representation of equation 7 fitted to the experimental data.
(TIF)

Figure S3 **Dependence of the change in the enzyme activity on the coverage of the hydrophobic transmembrane surface of PMCA by phospholipids.** The derivative of PMCA activity respect to θ_DPPC_ was calculated using equation 5 and the parameter values given in Table 1, and represented as a function of the enzyme activity (A), or θ_DPPC_ (B).
(TIF)

Figure S4 **Distribution of residuals after global fitting of the two-stage model.** The differences between the experimental data and the fitted values were calculated for: (A) the ATPase activity, Figure 1C, and (B) for apparent efficiency of energy transfer, Figure 4B.
(TIF)

Figure S5 **Relationship among the parameters of the minimal model and those of the two-stage model.** Activity data was numerically simulated for phospholipid mole fractions in the range 0–1 by using equation 8 and different sets of parameter values starting from those indicated in Table 1. Only one parameter was varied in each simulation series: *K*_ex_ (A and B), θ_0.5_ (C and D) and *c*_θ_ (E and F). Equation 9 was then fitted to the simulated data and the best fitting values of *X*_0.5_ (A, C and E) and *c*_x_ (B, D and F) were plotted as a function of the two-stage model parameter being explored in each series.
(TIF)
