# Supplementary figures and images for: A Two-Stage Model for Lipid Modulation of the Activity of Integral Membrane Proteins
Source: PLoS One. 2012 Jun 19;7(6):e39255. doi: 10.1371/journal.pone.0039255 (PMC3378530; doi:10.1371/journal.pone.0039255)

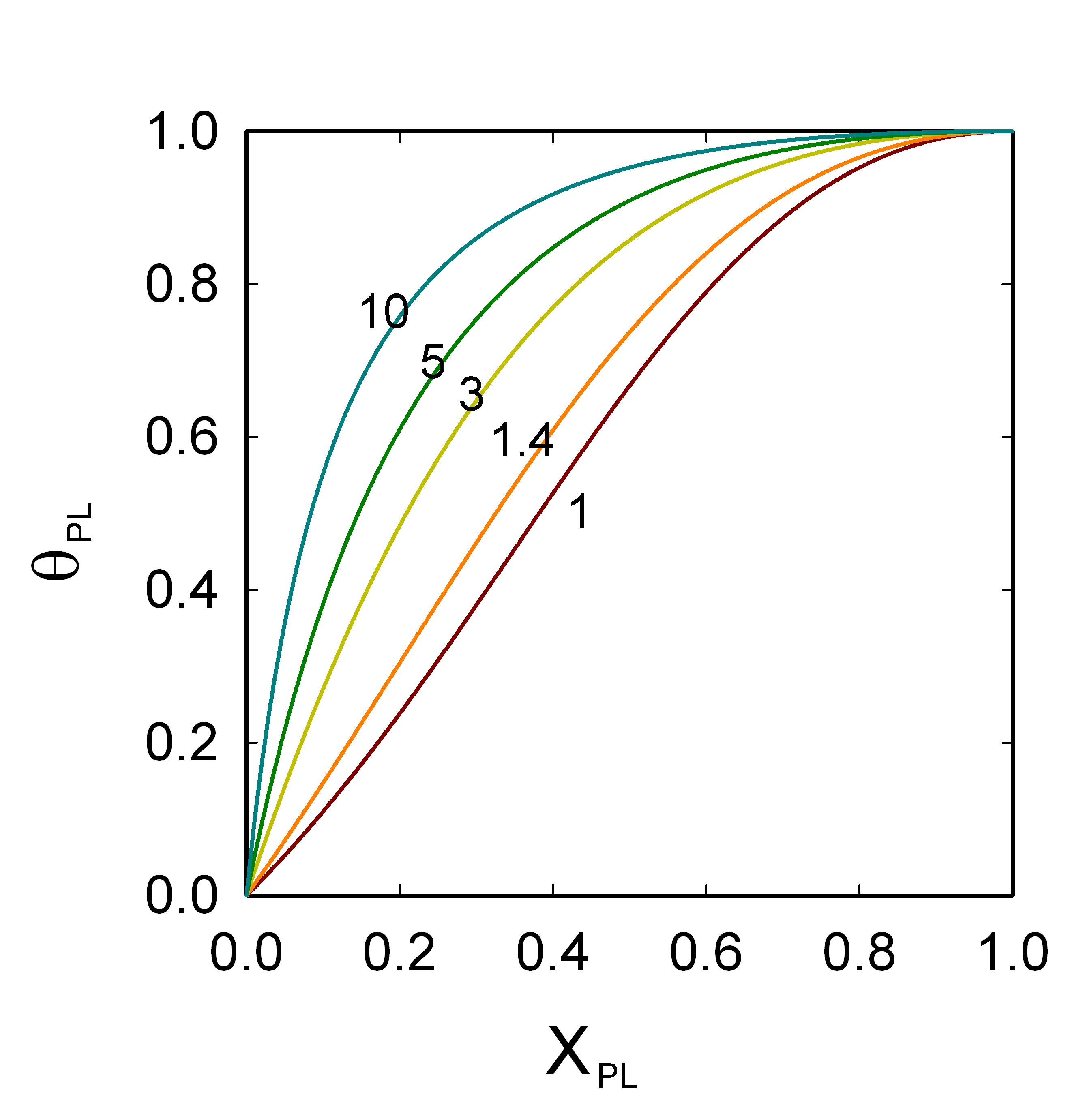

Supplement: Figure S1 — Dependence of the fractional coverage of the hydrophobic transmembrane surface of PMCA on the phospholipid mole fraction. Simulated values of the fractional coverage of the transmembrane surface by phospholipids (θPL) were obtained for the full range of micelle compositions using equation 2 and the K ex values indicated in the figure. The stoichiometric coefficient was taken equal to 2. The orange line corresponds to the K ex value determined in this work for the exchange DPPC/C12E10. (TIF) [file pone.0039255.s001.tif]
